# The efficacy and safety of amiodarone combined with beta-blockers in the maintenance of sinus rhythm for atrial fibrillation: A protocol for systematic review and network meta-analysis

**DOI:** 10.1097/MD.0000000000022368

**Published:** 2020-09-18

**Authors:** Shuqing Shi, Qiulei Jia, Jingjing Shi, Shuai Shi, Guozhen Yuan, Yuanhui Hu

**Affiliations:** a Cardiovascular Department, Guang’anmen Hospital, China Academy of Chinese Medical Sciences, Beijing, China; b Graduate School, Beijing University of Chinese Medicine, Beijing, China.

**Keywords:** amiodarone, atrial fibrillation, beta blockers, network meta-analysis, protocol, randomized controlled trials

## Abstract

**Background:**

The high recurrence rate of atrial fibrillation (AF) after recovering sinus rhythm has always been a clinical problem. Despite the established and widespread use of antiarrhythmic drugs, which one is better for maintaining sinus rhythm is still controversial. This study aims to summarize the randomized controlled trials (RCTs) of amiodarone combined with beta blockers to maintain sinus rhythm in AF, and to determine an effective and safe intervention for the prevention of AF recurrence through network meta-analysis (NMA).

**Methods and analysis:**

A comprehensive search of the RCTs comparing amiodarone with different beta-blockers to maintain sinus rhythm of AF patients will be conducted from the inception to December 2019 in the Cochrane Library, PubMed, Web of Science, EMBASE, Chinese Biomedical Literature Database (SinoMed), Chinese National Knowledge Infrastructure (CNKI), and WanFang database. The primary outcomes will be the recurrence of AF and frequency of embolization complications. The secondary outcomes will be the symptom improvements and adverse events. Risk of bias assessment of the included RCTs will be conducted according to the Cochrane collaboration's risk of bias tool. Pairwise meta-analyses and Bayesian network meta-analyses will be performed for all related outcome measures. GRADE will be used to evaluate the quality of evidence.

**Results:**

The results of this NMA will be published in a peer-reviewed journal.

**Conclusion:**

This NMA may provide more recommendations for patients and researchers, such as which treatment is better for a particular case of AF, and what may be the hotspots for the future studies.

**PROSPERO registration number:**

The protocol for this NMA has been registered on PROSPERO under the number CRD42020164438.

## 1 Introduction

Atrial fibrillation (AF) is the most common clinical arrhythmia. As the population ages, its morbidity and mortality are on the rise,^[1]^ which brings a heavy economic burden to the society and affects the patients’ quality of life.^[2–4]^ Until 2016, there were more than 33.5 million AF patients worldwide, with a population incidence of 2.5% to 3.5%.^[5]^ The main complications of AF include stroke, thromboembolism, myocardial infarction, heart failure, cognitive decline, dementia, and renal impairment, and the main treatment are ventricular rate control, rhythm control, and anticoagulant therapy, which are supported by the American Heart Association,^[6]^ European Society of Cardiology,^[4]^ and Canadian Cardiovascular Society.^[7]^ Rhythm control as a primary therapy can reduce the risk of thromboembolism and the usage of anticoagulant drugs, and improve the left ventricular function, the hemodynamics, patient symptoms, and their quality of life.

A major problem in the treatment of AF is the high recurrence rate. The risk of recurrence depends on age, AF duration, and the existence and severity of underlying heart disease.^[8,9]^ The overall rate of AF recurrence without treatment is high. In the patients who have converted to sinus rhythm, only 20% to 30% could remain in sinus rhythm 1 year later.^[10,11]^ Rhythm control in the early stage of AF is helpful to delay the progression and reduce the occurrence of AF-related complications,^[12]^ which can be regarded as the first choice for the treatment of recent-onset AF patients, paroxysmal AF patients, failed ventricular rate control patients, and the young patients who cannot tolerate AF symptoms.^[13,14]^

Long-term antiarrhythmic therapy has been widely used to prevent the recurrence of AF. Amiodarone is more effective than other antiarrhythmic drugs in maintaining sinus rhythm^[4]^; compared with Class I antiarrhythmic drugs, it has fewer adverse reactions.^[15]^ Beta-blockers are less effective than Class I or Class II antiarrhythmic agents in maintaining sinus rhythm, but their long-term adverse effects are significantly less than Class I or Class II antiarrhythmic agents. Therefore, amiodarone combined with beta-blockers are often used clinically to maintain sinus rhythm.^[16,17]^ However, current guidelines^[4,6,7]^ do not uniformly agree upon the recommendation of antidysrhythmic agents for maintenance sinus rhythm in AF, and drug preference in clinical practice also varies internationally.^[18,19]^ Previous systematic reviews and meta-analyses^[20,21]^ are limited by trials with different antiarrhythmic drugs and different treatment duration were pooled together; except for maintaining sinus rhythm, other results were not evaluated; and head-to-head drug comparisons are insufficient. Therefore, we intend to conduct a network meta-analysis (NMA) to compare and rank the efficacy and safety of amiodarone combined with different types of beta-blockers for maintaining sinus rhythm in AF patients.

## 2 Material and methods

### 2.1 Study registration

We will perform this systematic review and NMA according to the Preferred Reporting Items for Systematic Reviews and Network Meta-Analysis (PRISMA-NMA) statement.^[20]^ The protocol for this systematic review has been registered in PROSPERO with number CRD 42020164438.

### 2.2 Data sources and search strategy

We will do a comprehensive search of the Cochrane Library, PubMed, Web of Science, EMBASE, Chinese Biomedical Literature Database (SinoMed), Chinese National Knowledge Infrastructure (CNKI), and WanFang database from the inception to December 2019. In addition, we will search the bibliography of the included studies, as well as previous relevant systematic reviews, to look for other studies that may not be found by our database search. The search strategy will be adjusted according to the characteristics of different databases. Table 1 summarizes the details of the search strategy in PubMed.

**Table 1:**
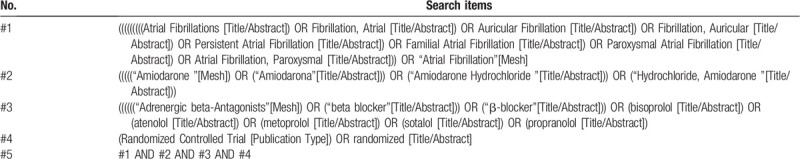
The search strategy in PubMed.

### 2.3 Inclusion and exclusion criteria

#### 2.3.1 Types of studies

RCTs about amiodarone combined with beta-blockers (bisoprolol, atenolol, metoprolol, sotalol, propranolol) for the maintenance of sinus rhythm of AF will be included without language restriction.

#### 2.3.2 Types of participants

Adults (>18 years) who have any type of AF and in whom sinus rhythm had been restored.

#### 2.3.3 Types of interventions

The included studies should randomly assign patients to the intervention group and the control group. The intervention group should receive long-term oral treatment with amiodarone combined with a certain β-blocker at an appropriate dose and aim at preventing the recurrence of AF and maintaining sinus rhythm.

#### 2.3.4 Types of comparison

The control group will be given amiodarone alone, or a placebo.

#### 2.3.5 Types of outcomes

Primary outcomes are as follows:

(1) The recurrence of AF (number of patients who have a recurrence of AF during the follow up, number of recurrences per patient) will be measured by electrocardiogram or 24 hours dynamic electrocardiogram.
(2) The frequency of embolization complications (stroke combined with peripheral embolism).

Secondary outcomes are as follows:

(1) Symptom improvements, including palpitations, chest tightness, dizziness, blackness, fatigue, and other symptoms.
(2) Adverse events, as reported by the individual trials: cardiac arrest, ventricular dysrhythmia, atrial flutter with 1:1 atrioventricular conduction, hypotension, and bradycardia.

### 2.4 Studies selection and data extraction

**Figure 1:**
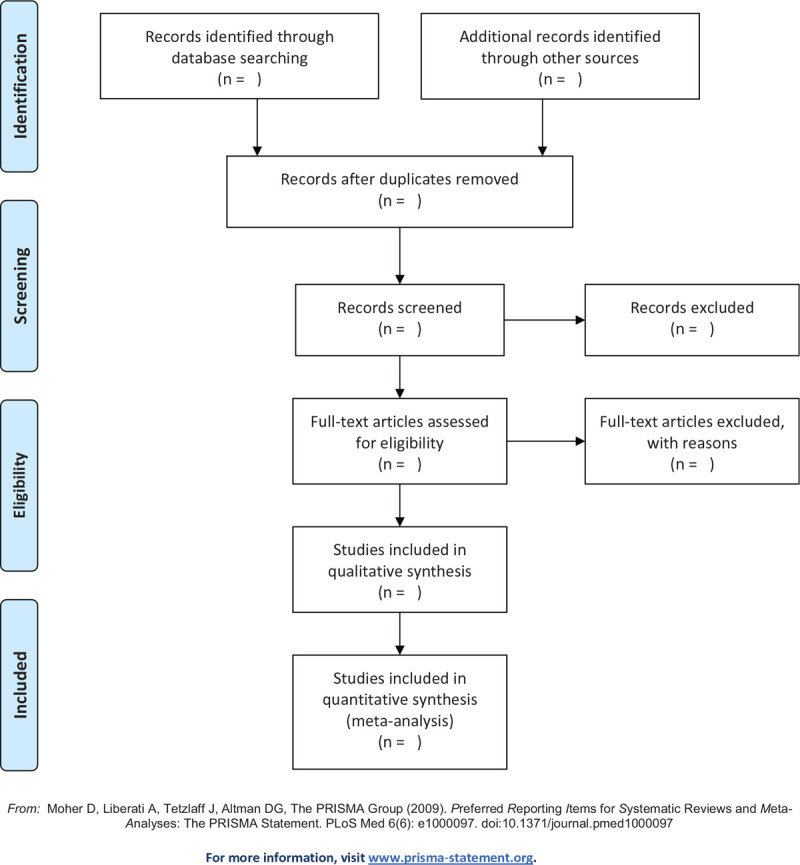
Flow diagram of studies search and selection.

All the retrieved titles and abstracts will be read and selected by 3 authors (SSQ, JQL, and SJJ) independently based on the inclusion and exclusion criteria. For the studies that initially meet the selection criteria, we will read the full text to determine their eligibility. In addition, the search of the references of the selected studies will be also conducted. The inconsistency between the 3 authors will be finalized by the corresponding author (YHH). The literature retrieval of this study will be conducted by the PRISMA flowchart, as shown in Figure 1. For each excluded study, the reason for the exclusion will be given.

Two authors (SS and GZY) will perform the data extraction independently. Standardized electronic data collection forms implemented by Microsoft Excel will be used to extract the following data: study design, diagnosis, number of participants, intervention types, outcomes, results, risk of bias assessment, and main conclusions.

### 2.5 Risk of bias assessment

The risk of bias of all included studies will be assessed independently by the Cochrane review handbook.^[22]^ The risk of bias tool includes 6 domains of bias: selection bias, performance bias, detection bias, attrition bias, reporting bias, and other bias. All divergences will be solved by consensus. Each study will be classified as high or low risk in each domain at the study level and outcome (conversion to sinus rhythm) level.

### 2.6 Data synthesis and analysis

#### 2.6.1 Pairwise meta-analysis

If there are 2 or more RCTs that meet the inclusion criteria and report the same outcomes in comparable populations, we will use a random effects model to conduct a pairwise meta-analysis for each treatment. The standard pairwise meta-analysis will be performed by Stata V.14.0 software. Risk ratio (RR) and 95% credibility interval (CI) will be used for dichotomous outcomes, mean differences, or standard mean differences (SMDs) with 95% CI will be used for continuous outcomes. Clinical and methodological heterogeneity of the trials will be assessed by the χ^2^ test and *I*^2^ statistic. The sources of heterogeneity will be explored by subgroup analysis and sensitivity analyses.^[23]^

#### 2.6.2 Assessment of inconsistency

Consistency check is one of the major assumptions that NMA relies on.^[24]^ We will use node-split approach to contrast the direct evidence with the indirect evidence on each node of the network when we assess the evidence for consistency in the networks.

#### 2.6.3 Network meta-analysis

We will complete the NMA by using Stata V.14.0 and WinBUGS 1.4.3 (Medical

Research Council Biostatistics Unit, Cambridge, UK).^[25]^ With the data extracted from the included studies, we will create a network diagram to illustrate which treatments (nodes) are directly compared and which are indirectly compared through one or more general intermediaries. A complex statistical model will be built by a Bayesian software program, and the Markov Chains Monte Carlo simulation technique will be used to generate samples. The initial burn-in period will be set to 5000 iterations for convergence, then the posterior summaries will be set to a further 30,000 iterations.^[26]^ In addition, the surface under the cumulative ranking curve (SUCRA) will be presented as well to rank the probabilities of all prevention interventions for each outcome.^[27]^

#### 2.6.4 Subgroup and sensitivity analyses

We will perform subgroup analyses according to age (age ≤60 and >60 years), gender (male and female), AF types (paroxysmal AF, persistent AF, and permanent AF), and location (general ward and intensive care unit). Furthermore, we will conduct a sensitivity analysis to check each study sequentially and combine the remaining studies to identify the impact of each study on the overall outcome.

#### 2.6.5 Quality of evidence

In order to assess the treatment quality of NMA, the assessment of the direct, indirect, and mixed evidence is necessary, as well as quality ratings for direct and indirect comparisons.^[28]^ According to the GRADE working group recommendations, we will assess the evidence quality to estimate the AF treatment effect of NMA.^[29]^ We will present the direct and indirect treatment estimates and the NMA estimate for each comparison of the evidence network.^[30]^

### 2.7 Ethics and dissemination

It is not necessary for ethical approval because it is based on published studies. The protocol will be disseminated in a peer-reviewed journal or presented at a topic-related conference.

## 3 Discussion

This systematic review will provide updated evidence for amiodarone combined with beta blockers to maintain sinus heart rate in patients with AF. According to a Cochrane systematic review,^[9]^ amiodarone showed some advantages over the Class I and other Class III drugs with 1-year follow-up. Amiodarone is a multi-ion channel blocker with anti-arrhythmic effects. It reduces the autonomy and conductivity of autonomous cells by blocking the I_Na_, I_Kr_, and L-type calcium channels.^[31]^ It is more effective in preventing the recurrence of AF, and it does not produce obvious pre-arrhythmia and increase the mortality. However, amiodarone has significantly more adverse reactions than placebo, and it is well known that its adverse reactions frequency increase with time. Therefore, clinically, amiodarone is often used in conjunction with beta-blockers to increase efficacy and safety. To the best of our knowledge, this will be the first systematic review concerning this topic. Considering the importance of maintaining sinus rhythm in the treatment of AF, we hope this systematic review can provide the health care workers with more high-quality evidence to choose antiarrhythmic drugs, amiodarone, and β-blockers for patients with AF.

## Author contributions

**Conceptualization:** Shuqing Shi, Jingjing Shi.

**Data curation:** Shuqing Shi, QiuleiJia

**Methodology:** Shuai Shi, Guozhen Yuan

**Project administration:** Yuanhui Hu

**Writing – original draft:** Shuqing Shi, Jingjing Shi

**Writing – review & editing:** Shuqing Shi, Qiulei Jia.

## Abbreviations

Abbreviations: AF = atrial fibrillation, CNKI = Chinese National Knowledge Infrastructure, NMA = network meta-analysis, RCT = randomized controlled trial.
